# Macroscopic Appearance of Giant Adrenal Myelolipoma During Laparoscopy: An Adjunct in Differential Diagnosis

**DOI:** 10.7759/cureus.6582

**Published:** 2020-01-07

**Authors:** Antonios Katsimantas, Dimitrios Filippou, Argiro Melloy, Spyridon Paparidis, Nikolaos Ferakis

**Affiliations:** 1 Urology, Mediterraneo Hospital, Glyfada, GRC; 2 Surgery, National and Kapodistrian University of Athens School of Medicine, Athens, GRC; 3 Histopathology, General Hospital of the Greek Red Cross "Korgialeneio-Benakeio", Athens, GRC; 4 Urology, Korgialenio-Benakio Hellenic Red Cross Hospital, Athens, GRC

**Keywords:** adrenal myelolipoma, laparoscopy, retroperitoneal tumor

## Abstract

Giant adrenal myelolipoma is a rare, benign, sizable, mesenchymal tumor. Preoperative differential diagnosis from retroperitoneal liposarcoma may be challenging. A 66-year-old female patient was admitted because of a sizable tumor at the right retroperitoneal space, incidentally discovered during abdominal ultrasonography for screening purpose. Preoperative imaging studies were indicative for the diagnosis of a giant adrenal myelolipoma (11.7 × 12.9 cm in size); however, a retroperitoneal liposarcoma could not be excluded. We decided to proceed with tumor’s surgical removal by using laparoscopic transperitoneal approach and three-dimensional high-definition camera. Intraoperatively, the tumor did not infiltrate surrounding tissues and was surrounded by a thin capsule under which there were sparse, orange-colored spots that resembled adrenal cortex. This finding reinforced the initial and most possible diagnosis of adrenal myelolipoma and we easily enucleated the mass. Postoperative course was uneventful, and the patient demonstrated no recurrence on imaging six months postoperatively. Histology confirmed the diagnosis of giant adrenal myelolipoma, measuring 16.5 x 15 x 6.5 cm.

## Introduction

Adrenal myelolipomas (AMLs) are rare, benign, non-secretory, mesenchymal tumors, composed of mature adipose and myeloid tissue. Size of the tumor is usually less than 4 cm, but it can outreach 10 cm and in this case the tumor is characterized as giant. The diagnosis of AML is indicated by clinical presentation, although it is usually asymptomatic, and by imaging studies and is confirmed by pathological examination. The treatment of choice of giant AML is open surgical removal, but there are numerous reports of giant AMLs which were successfully removed by using minimal invasive approaches (pure laparoscopy, robotic-assisted laparoscopy) during the last years [1].

The aim of our study is to present the macroscopic appearance of a giant AML as it was recognized intraoperatively during the laparoscopic excision and to underline the feasibility of successful removal of this kind of tumors by using minimal invasive approaches.

## Case presentation

In February 2019, a 66-year-old female patient was admitted to the Department of Urology of Korgialenio-Benakio Hellenic Red Cross Hospital, Athens, Greece, because of a sizable tumor at the right retroperitoneal space, incidentally discovered during abdominal ultrasonography (U/S) for screening purpose two weeks ago. Her medical history included hypertension, dyslipidemia and hypothyroidism. The patient was asymptomatic, clinical examination was unremarkable, while blood chemistry and adrenal hormones were in the normal range.

**Figure 1:**
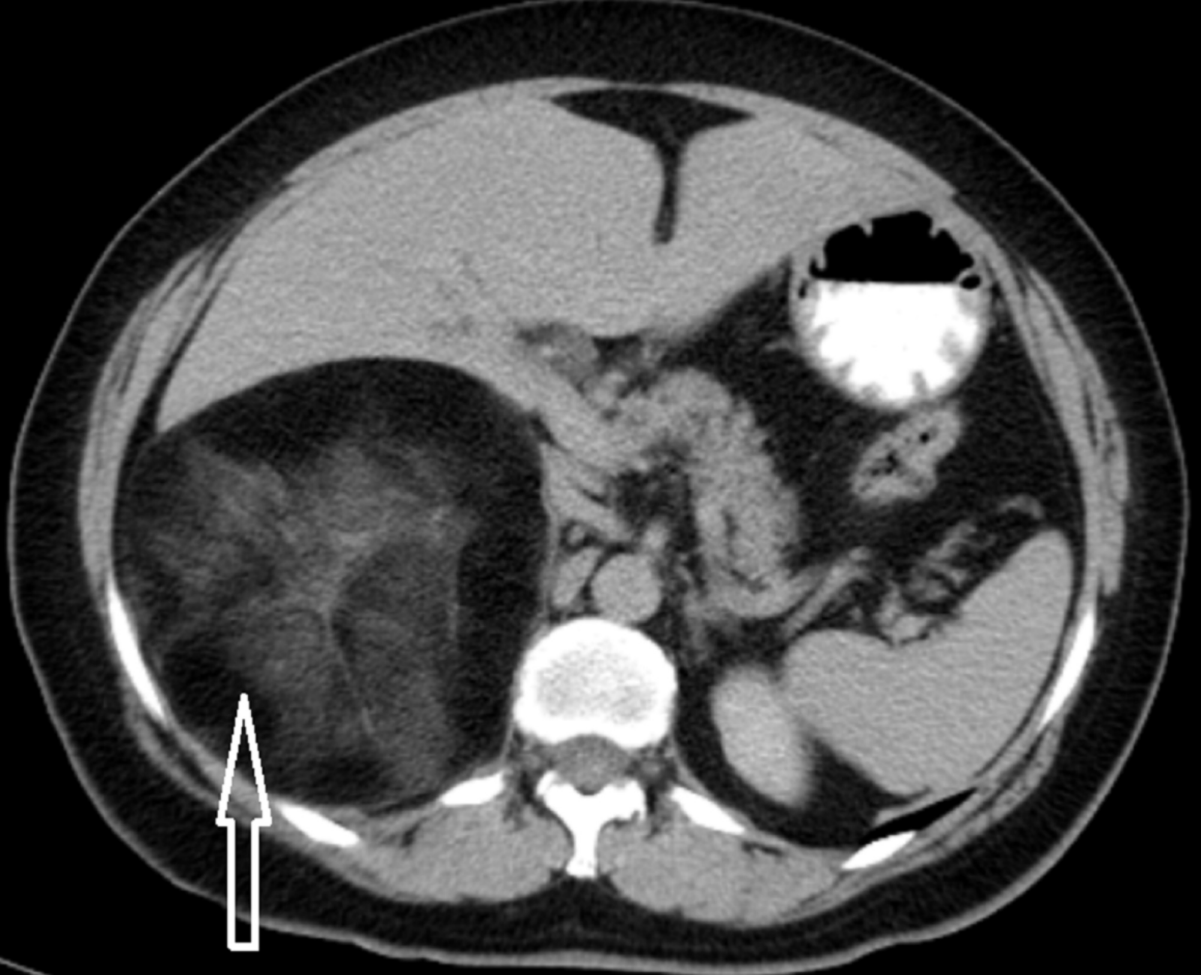
Preoperative abdominal CT scan demonstrating a 12.9 × 11.7 cm in size, encapsulated, hypodense, solid, right retroperitoneal mass (arrow), containing fat (mainly), interspersed soft-tissue attenuation components and a few septa. The tumor displaced liver’s right lobe anteriorly.

Preoperative U/S described the presence of a heterogeneous, solid, 11 cm in size mass below liver’s right lobe. Preoperative abdominal computed tomography (CT) scan demonstrated a 12.9 × 11.7 cm in size, encapsulated, hypodense, solid, right retroperitoneal mass, containing mainly fat as well as interspersed soft-tissue attenuation components and a few septa. The tumor displaced liver’s right lobe anteriorly and right kidney caudally, while the right adrenal gland was not identified in its normal anatomical position (Figure 1). After administration of contrast medium, the lesion exhibited poor enhancement with a rapid washout. Magnetic resonance imaging (MRI) confirmed the presence of a mass consisting of fat and solid elements. The lesion had hyperintense signal on T1-weighted sequences, isointense signal on T2-weighted sequences and hypointense signal on fat-suppression sequences. Based on preoperative evaluation, the most possible diagnosis was AML as the tumor was well defined, with a recognizable capsule, compressing the surrounding tissues rather than infiltrating them. However, we could not exclude retroperitoneal liposarcoma due to the tumor’s size and the presence of concomitant solid pattern.

**Figure 2:**
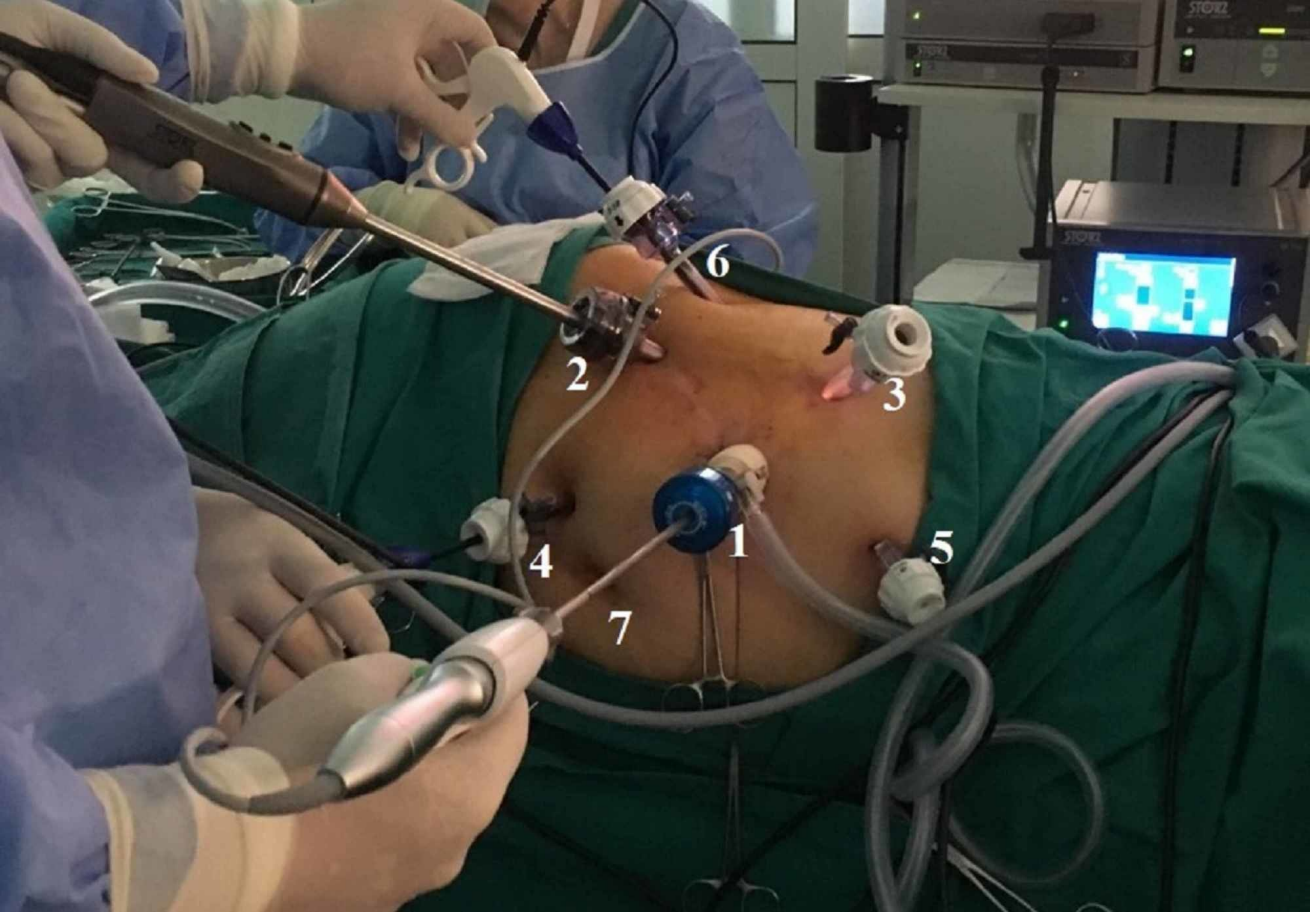
Intraoperative photo demonstrating port placement for right laparoscopic transperitoneal tumor resection. Each number corresponds to the points of trocars’ placement: 12 mm Hasson trocar for the 30° laparoscope (1), 12 mm surgeon’s trocar (2), 10 mm surgeon’s trocar (3), 5 mm assistant’s trocar (4), 5 mm subxyphoid trocar (5) and 10 mm trocar which was decided to be placed intraoperatively in order to approach the posterior surface of the tumor (6). Umbilicus (7).

**Figure 3:**
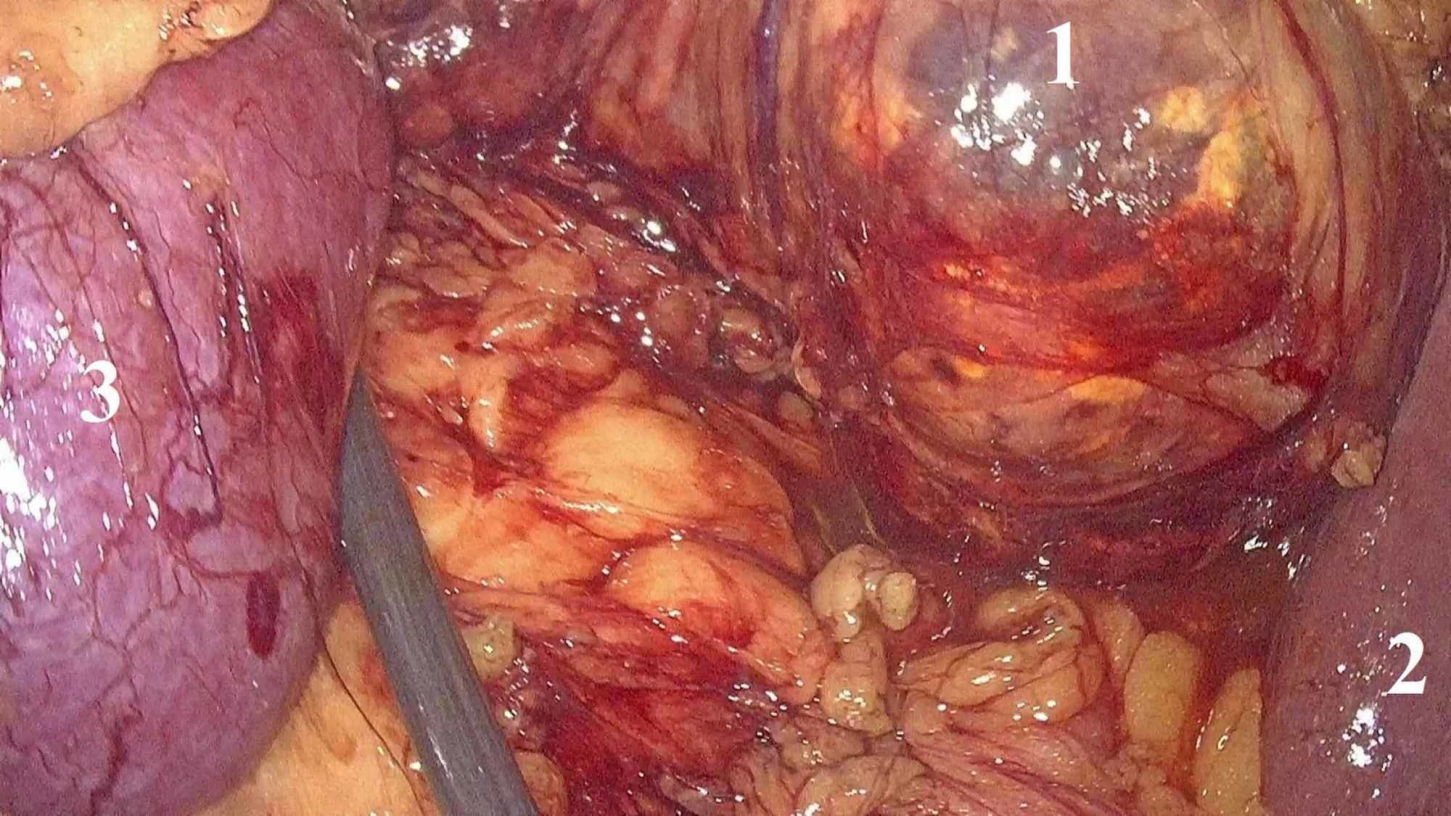
Intraoperative photo demonstrating the tumor (1) between the liver’s right lobe (2) and right kidney’s upper pole (3). The tumor (1) was surrounded by a thin capsule under which there were sparse, orange-colored spots that resembled adrenal cortex.

We decided to proceed with tumor’s surgical removal after obtaining informed written consent from the patient. Our approach was laparoscopic lateral transperitoneal, under general anesthesia. A three-dimensional high-definition camera was used, and the configuration and numbering of trocars are demonstrated in Figure 2. An open Hasson technique was used in order to place the first trocar (trocar No 1) of 12 mm for the 30° laparoscope four fingerbreadths above and 8-10 cm lateral to the umbilicus. The rest of trocars were placed under direct vision as follows: one trocar of 12 mm (trocar No 2) and one trocar of 10 mm (trocar No 3) at the midclavicular line according to the triangulation principle, serving as surgeon’s working channels; one trocar of 5 mm (trocar No 4) one fingerbreadth below and two fingerbreadths lateral to the umbilicus for the first assistant; one subxyphoid trocar of 5 mm (trocar No 5) to retract liver. The colon's lateral attachments and hepatorenal ligaments were released. Duodenum was then mobilized medially (Kocher maneuver), and vena cava was clearly visualized. Gerota’s fascia was opened, and the genital vessels, the proximal ureter, the psoas muscle and the renal pedicle were located. Following Gerota’s incision, the tumor was recognized between the liver’s right lobe and right kidney’s upper pole. The tumor was soft in texture, did not infiltrate surrounding tissues and was surrounded by a thin capsule under which there were sparse, orange-colored spots that resembled adrenal cortex (Figure 3). Tumor’s appearance reinforced the initial diagnosis of AML, and the mass was easily enucleated following its capsule. Intraoperatively, another 10 mm trocar (trocar No 6) was placed at the posterior axillary line, two fingerbreadths below umbilicus, which was used as surgeon’s working channel, and the laparoscope was placed in trocar No 2 to approach the posterior surface of the tumor. The tumor was removed by expanding the initial incision for the trocar No 2 by using an endoscopic specimen bag. Operative time was 146 minutes, and no intraoperative complications were encountered.

**Figure 4:**
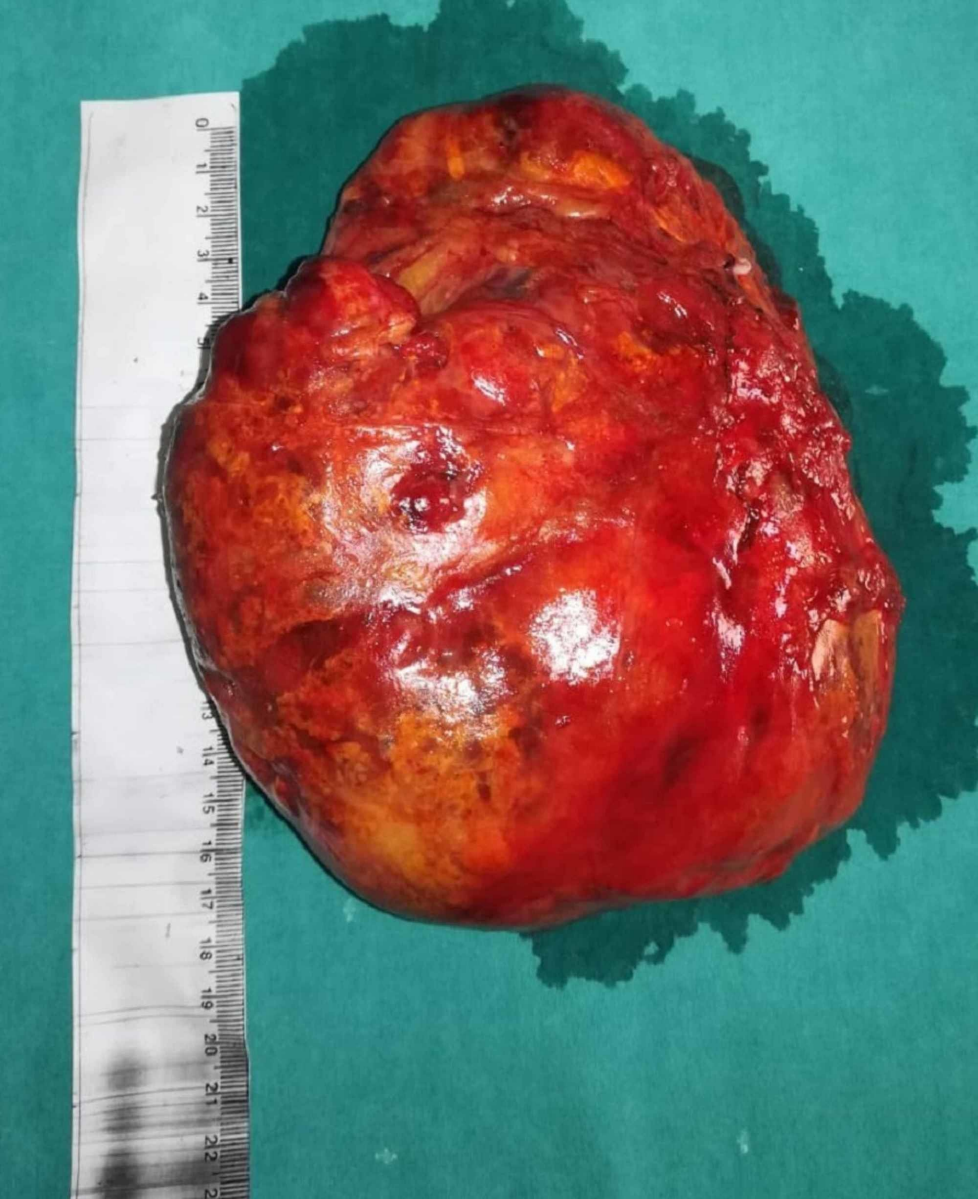
Postoperative photo demonstrating the surgical specimen.

**Figure 5:**
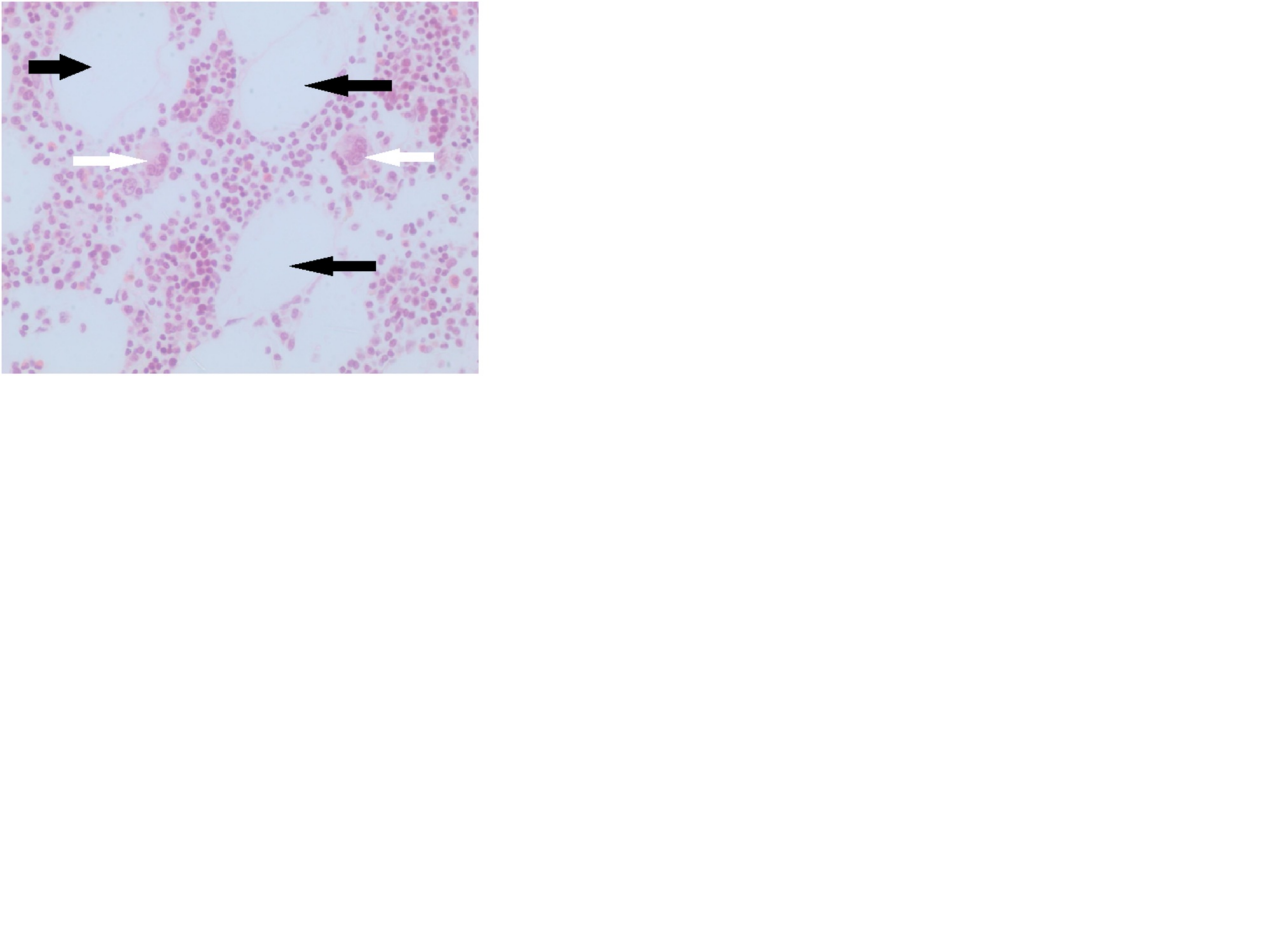
Histological (microscopical) image showing characteristics of adrenal myelolipoma with mature fat (black arrows) and hematopoietic elements (white arrows) (hematoxylin and eosin stain, ×400).

**Figure 6:**
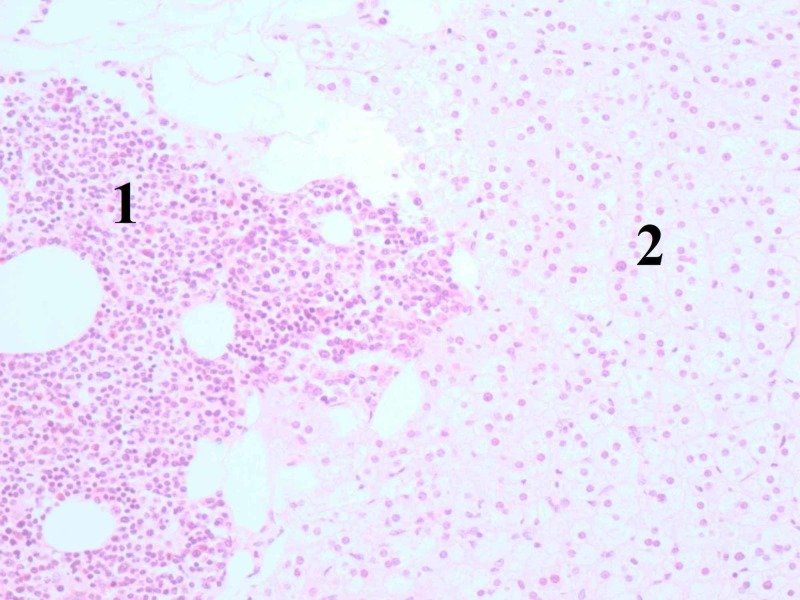
Histological (microscopical) image demonstating myeloid tissue (1) in contact with normal adrenal tissue (2) (hematoxylin and eosin stain, ×200).

Macroscopic examination revealed a gray-yellow, encapsulated, irregularly shaped mass, 16.5 x 15 x 6.5 cm in size (Figure 4). On cut section, the tumor had fatty appearance and elastic texture. Microscopically, the tumor consisted of mature adipocytes admixed with hematopoietic components, including myeloid, erythroid and megakaryocytic elements (Figure 5). On tumor’s total periphery, there were sites of compressed, normal adrenal tissue, and this finding was similar to the intraoperative appearance of the tumor (Figure 6). These findings confirmed the diagnosis of AML.

The patient had an uneventful postoperative course and was discharged on the third postoperative day. The patient is followed up on an outpatient basis, and she demonstrated no recurrence on imaging six months postoperatively.

## Discussion

AMLs are rare, benign tumors composed of mature adipose tissue and bone marrow represented by all three lineages of hematopoietic elements [1,2]. Their incidence is 0.08%-0.4% at autopsy, and they represent 10%-15% of adrenal incidentalomas [1,3]. Their size varies and is usually less than 4 cm. They usually occur between fifth to seventh decades of life, with no gender or site predilection [1,2]. They are usually encountered unilaterally, although they can occur bilaterally or outside adrenal gland [4]. Giant AMLs, exceeding 10 cm in diameter, are even rarer [2].

The origin and pathogenesis of AMLs are unclear [1,3]. The risk factors include infection, inflammation, necrosis, stressful lifestyle, obesity, hypertension, diabetes and unbalanced diet [1]. They may coexist with hormone-secreting adrenal masses, but AMLs are always hormonally inactive [2]. Theories about their pathogenesis include reticuloendothelial cell metaplasia of adrenal capillaries, emboli from bone marrow and adrenal embryonic remnants of hematopoietic elements, while there is a report of myelolipoma expressing (3;21) (q25;p11) translocation which indicates a neoplastic process of hematologic origin [5].

AMLs are usually asymptomatic and incidentally identified during imaging studies presented for other reasons, as they are usually small, located retroperitoneally and hormonally inactive [1,2]. Larger tumors may be palpable or may cause symptoms related to compression of surrounding tissues, which were not confirmed in our case [6,7]. In addition, larger tumors may be complicated by spontaneous rupture and bleeding, manifesting with pain and hemodynamic shock [1,2].

As observed in our case, AMLs are presented as hyperechoic or hypoechoic images on U/S, depending on the predominance of fatty tissue or bone marrow elements, respectively [8]. On CT scan, they are demonstrated as low-attenuation, well-defined, encapsulated lesions containing fat density along with higher density myeloid elements, as in our case [2,3]. Punctuate calcifications and hemorrhagic areas can be encountered in some cases [2]. On MRI, tumor’s fat appears typically hyperintense on T1-weighted sequences which is confirmed on fat-saturation sequences, while bone marrow component has low and moderate signal on T1-weighted and T2-weighted sequences, respectively [9,10]. Definite diagnosis is based on histology which reveals mature adipose tissue and hematopoietic elements representing all of the three hematopoietic lineages (granulocytic, erythroid, megakaryocytic), consistent to our findings [10].

Lesions like adrenal adenoma or carcinoma, metastases, pheochromocytoma and renal angiomyelolipoma are usually excluded based on preoperative investigation [11]. Extramedullary hematopoiesis is easily excluded, as it is associated with anemia, hepatosplenomegaly and infiltration of bone marrow by lymphoma/leukemia cells [8]. Differential diagnosis between AML and retroperitoneal liposarcoma can be challenging in cases of large tumors, with high volume of hematopoietic component, as observed in our present case. Recently, ^111^indium chloride bone marrow scan has been proposed as an adjunct in preoperative evaluation in such cases. In our case, we decided to approach the tumor as benign, based on preoperative imaging studies which demonstrated a lipid-rich, well-defined, encapsulated mass, which did not infiltrate or change the borders of surrounding tissues. The endoscopic appearance of the tumor during laparoscopy and the fact that it was easily dissected reinforced our initial planning and we did not proceed with wide resection, which is demanded in case of retroperitoneal liposarcoma [12]. Finally, both endoscopic appearance and final histology demonstrated sites of normal adrenal tissue on tumor’s periphery.

AMLs demonstrate no malignant potential, and active surveillance with regular imaging follow-up is the appropriate treatment for small, asymptomatic lesions [3]. Surgical resection is recommended in cases where the diagnosis is equivocal or in tumors larger than 6 cm which are usually accompanied by symptoms or are at high risk for complications [1]. Giant myelolipomas are at high risk of spontaneous rupture and bleeding [2]. Open surgical removal is the approach of choice for giant AMLs, while there are only a few reports for tumor’s removal by using minimal invasive approaches [1]. Our case confirms that giant AMLs can be safely removed by using minimally invasive surgical means. We believe that laparoscopy should be preferred provided that the necessary equipment is available and the surgeon is experienced, as minimal invasive techniques outperform conventional ones in terms of postoperative pain, length of hospitalization and cosmesis [1,4]. In addition, optical magnification of laparoscopy perhaps contributed in the adequate identification of adrenal tissue below tumor’s capsule intraoperatively, which guided our decision to proceed with enucleation of the mass instead of performing a wide resection.

## Conclusions

Preoperative diagnosis of giant AML can be difficult as it resembles retroperitoneal liposarcoma. However, the surgical approach between these clinical entities differs significantly. The macroscopic appearance of the tumor during laparoscopy guided our decision to avoid wide resection and the final histological findings were concordant with the intraoperative ones. Giant AMLs can be surgically removed efficiently and safely by using the laparoscopic approach.
